# Artificial intelligence for alcohol, opioid, and cannabis use disorders screening and management: a narrative review of barriers and facilitators to clinical implementation

**DOI:** 10.3389/fdgth.2026.1958597

**Published:** 2026-09-21

**Authors:** Isaac Frimpong, Benedictus Atsu Hlormenu

**Affiliations:** 1 Department of Public Health, University of California, Berkeley, CA, United States; 2 School of Nursing and Health Studies, University of Miami, Coral Gables, FL, United States

**Keywords:** artificial intelligence, machine learning, substance use disorder, implementation science, clinical decision support, relapse prevention, algorithmic fairness, digital health

## Abstract

Substance use disorder (SUD) remains one of the most prevalent and undertreated conditions in medicine. Machine learning and artificial intelligence (AI) have produced numerous predictive models for SUD risk stratification, screening, and management, but few have progressed beyond development and validation into sustained clinical implementation. This review focuses on alcohol, opioid, and cannabis use disorders, the substances for which a deployed or near-deployed AI evidence base currently exists, synthesizing barriers and facilitators to AI implementation using a hybrid framework integrating the Framework for AI Implementation Research in Healthcare (FAIIR-H) with the Unified Theory of Acceptance and Use of Technology (UTAUT) across four domains: data and model, clinician and workflow, patient, and system and regulatory factors. Alcohol use disorder has the largest predictive literature by volume but remains methodologically heterogeneous with limited external validation; opioid use disorder has a smaller but more methodologically mature and fairness-audited literature; cannabis use disorder has a more limited evidence base. Two real-world deployments illustrate this gap being bridged, with differing strength of evidence: a hospital-based opioid AI screener, supported by fairness-auditing and implementation-outcome evidence, was associated, as a secondary pre–post finding, with 47% lower odds of 30-day readmission across more than 51,000 hospitalizations; an alcohol relapse-management platform was associated with up to an 18% reduction in relapse risk within a platform dataset of more than 500,000 patient-days of observational data, though comparable implementation-outcome and fairness evidence was not identified for it. Among the studies identified in this review, no substance has yet demonstrated success in both screening and management simultaneously. Implementation is further shaped by stigma, data-sharing concerns, digital access, and the SUD-specific confidentiality requirements of 42 CFR Part 2. We propose a three-tier typology of management-focused AI applications showing that patient trust burden escalates with directness of AI-patient interaction rather than predictive accuracy and outline a measurable research and policy agenda targeting external validation, fairness auditing, and implementation in safety-net settings.

## 1 Introduction

Substance use disorders remain among the most prevalent and undertreated conditions in medicine. The United Nations Office on Drugs and Crime estimated that 316 million people used drugs globally in 2023, a 28 percent increase over the preceding decade, with approximately 64 million people affected by drug use disorders and only an estimated 8.1 percent accessing treatment (1). Among hospitalized adults, substance-related problems affect an estimated 15 to 25 percent of patients, yet fewer than half of cases are detected through routine screening, and fewer than one quarter of identified patients receive evidence-based treatment before discharge (2). Much of this gap traces to the tools used for detection itself. Traditional screening approaches, including unstructured clinical judgment, patient self-report, and standardized questionnaires, remain heavily relied upon by healthcare professionals, but each carries recognized weaknesses: clinical judgment is inconsistent across providers and vulnerable to implicit bias, self-report is subject to underreporting driven by stigma and fear of consequence, and questionnaire-based screening depends on consistent administration that time-pressured clinical encounters often do not allow. Together these limitations contribute to missed diagnoses, reporting bias, and inconsistent implementation of screening protocols across settings, the gap into which AI-based tools have been proposed to intervene. This treatment gap spans the continuum of care. At the point of detection, Screening, Brief Intervention, and Referral to Treatment (SBIRT) programs remain the standard of care but are constrained by workforce limitations, time pressures, and inconsistent implementation (3, 4). After discharge, relapse rates of 40 to 60 percent within the first year highlight persistent challenges in long-term engagement and continuity of care (5).

Whether frontline addiction clinicians are prepared to adopt new technologies is not a new question. A 2019 survey of European health professionals identified substantial gaps in knowledge, confidence, and use of technology-based SUD interventions, prior to widespread discussion of AI-specific tools (6). The broader landscape of digital interventions for alcohol, opioid, and cannabis use disorders, including websites, apps, and SMS-based tools, has since expanded considerably, though a 2024 scoping review found substantial methodological heterogeneity across this literature (7). Hundreds of AI studies have focused on predictive accuracy and technical validation, leaving a gap in studies of implementation barriers that this review addresses.

AI has been proposed to close both gaps: on the screening side, by automating detection from clinical documentation, and on the management side, the less-explored focus of this review, through relapse and dropout prediction, treatment-outcome forecasting, and AI-generated recovery support. A digital phenotyping approach using social media language outperformed the Addiction Severity Index in predicting 90-day treatment outcomes (8). A 2025 study using deep learning on smartphone-based ecological momentary assessment data predicted non-prescribed opioid use and treatment retention with moderate to good accuracy, though performance was lower for medication non-adherence specifically (9). A federally funded protocol is evaluating how AI-generated recovery support messages for alcohol use disorder can be optimized for engagement, trust, and clinical benefit (10).

Previct Alcohol, a commercial eHealth platform in clinical use since late 2015, incorporated an AI-driven relapse-prediction component in May 2021. In an observational analysis spanning more than 500,000 patient-days, caregiver notification of a predicted relapse was associated with a 9% reduction in short-duration relapse risk and an 18% reduction in relapse risk for relapses lasting more than 3 days, relative to periods without caregiver notification (11).

This challenges the assumption that opioid screening is the only area of SUD care where AI has reached implementation. Instead, among the studies identified in this review, implementation remains asymmetric: opioid care provides the strongest real-world example of AI-enabled screening, alcohol care provides the strongest example of AI-enabled management, and no substance has yet demonstrated success across both. A close comparator, a 2025 narrative review mapping the technical landscape of digital therapeutics and AI-assisted relapse-prevention monitoring, characterizes existing tools but does not examine implementation factors (12); similarly, a 2024 review outlining broad challenges and opportunities for AI in addiction care addresses technical and ethical considerations without an implementation-science framework (13), the gap this review addresses. In this review, implementation is defined broadly across three dimensions: deployment within clinical workflows, clinician- and patient-facing acceptance and usability, and organizational and regulatory readiness. This approach recognizes implementation as a continuum rather than a single stage of translation.

This prediction-to-practice gap is not unique to alcohol, opioid, and cannabis use disorders. Across healthcare, growing numbers of AI models have not translated into routine use, prompting implementation-science scholarship applying the Consolidated Framework for Implementation Research (14) and broader barriers-and-facilitators syntheses (15). A 2025 scoping review of 46 studies on clinician AI acceptance, using the Unified Theory of Acceptance and Use of Technology (UTAUT), found performance expectancy and facilitating conditions functioning ambivalently as barriers or facilitators depending on context, and identified additional themes including clinician hesitancy, legal and ethical concerns, and limited low- and middle-income country representation (16). Similar findings appear in orthopaedic practice (17). These studies, together with the new Framework for Artificial Intelligence Implementation Research in Healthcare (FAIIR-H) (18), provide a methodological foundation not yet applied to SUD specifically.

The limited number of successful deployments illustrates the gap concretely. A hospital-based AI tool for opioid use disorder risk, implemented prospectively across more than 51,000 hospitalized adults, was associated, as a secondary outcome of a pre–post quasi-experimental evaluation, with a 47 percent lower odds of 30-day readmission and lower healthcare costs (2); as detailed in Section 5.1, this finding should be interpreted as an association rather than a demonstrated causal effect. This built on the previously validated SMART-AI deep learning algorithm (19). Previct Alcohol provides a complementary example for management (11). Together, these deployments show AI can be translated into routine SUD care, but only within a narrow set of applications.

This review synthesizes barriers and facilitators to implementing AI-based SUD screening and management tools using a hybrid framework across four domains: data and model-level, clinician and workflow-level, patient-level, and system and regulatory-level factors. We examine the two deployment cases above as illustrations of successful translation, extend the analysis to the broader but earlier-stage management literature, contrast findings with the largely undeployed cannabis literature, identify features that make SUD a distinct AI implementation context, situate findings within the broader implementation literature from fields such as ophthalmology and radiology, and propose a research and policy agenda.

## 2 Review methodology

### 2.1 Design

This review adopts a narrative rather than systematic approach, synthesizing a rapidly evolving, methodologically heterogeneous literature spanning clinical medicine, addiction science, implementation science, health informatics, and AI into a coherent conceptual framework rather than an exhaustive catalogue. The review follows the Scale for the Assessment of Narrative Review Articles (SANRA) reporting principles, including explicit justification of aims, a documented search strategy, and traceable referencing (20). Full Boolean search strings are provided in Supplementary File S1.

### 2.2 Conceptual framework

Two bodies of literature inform this review but have not previously been integrated. The first is the rapidly developing and increasingly theory-driven literature on barriers to AI implementation in healthcare, conducted largely outside addiction medicine; for example, Scipion et al. (16) synthesized 46 studies spanning ophthalmology, radiology, primary care, emergency medicine, and mental health, but none focused specifically on alcohol, opioid, and cannabis use disorders. The second is the longstanding, largely pre-AI literature on barriers to alcohol, opioid, and cannabis use disorders screening and treatment, which has consistently identified stigma, confidentiality concerns, workforce limitations, and fragmentation across care systems (3, 4, 6).

This review integrates both using a hybrid framework: FAIIR-H (18) as the macro-structure for data, model, organizational, and regulatory domains, and UTAUT constructs, performance expectancy, effort expectancy, social influence, and facilitating conditions (16, 21), within the clinician and workflow domain specifically, since UTAUT lacks native coverage of the domains

FAIIR-H addresses. To our knowledge, this hybrid framework has not previously been applied to SUD-specific AI implementation, offering a structured basis for distinguishing which general-healthcare AI implementation barriers apply to SUD care, which are amplified by SUD-specific characteristics, and which require additional attention entirely.

In this review, “largest” refers to literature volume, whereas “most mature” or “most advanced” refers to how far a substance's literature has progressed through the translation stages described below, particularly external validation and prospective evaluation, as well as the extent of subgroup fairness evaluation reported. A literature can be large without being methodologically mature, and we treat these as separate dimensions throughout.

We also distinguish six stages of AI translation: model development, internal validation, external validation, prospective evaluation, live clinical deployment, and sustained implementation. Each study is classified according to the highest stage for which evidence was reported. Throughout, we further distinguish three levels of evidence: predictive performance, clinical effectiveness, and implementation outcomes. Predictive performance refers to model discrimination, calibration, and subgroup fairness; clinical effectiveness refers to patient- or service-level outcomes such as readmission, relapse, or treatment initiation; and implementation outcomes refer to constructs such as acceptability, adoption, appropriateness, feasibility, fidelity, penetration, implementation cost, and sustainability. “Successful implementation” refers specifically to evidence of relevant implementation outcomes, such as acceptability, adoption, feasibility, fidelity, or sustainability, rather than clinical effectiveness alone. Thus, a reduction in readmission or relapse rates may indicate clinical benefit but does not, by itself, establish implementation success.

### 2.3 Search strategy

Structured searches of PubMed, Embase, Web of Science, and PsycINFO were supplemented by citation tracking and targeted preprint-server searches (medRxiv and arXiv) given the pace of AI publication. Search terms combined AI-related concepts (artificial intelligence, machine learning, deep learning) with substance-specific concepts (substance use disorder, opioid, alcohol, cannabis) and implementation-related terms (implementation, barriers, facilitators, adoption, acceptance, deployment). In PubMed, Medical Subject Headings (MeSH) were combined with free-text title/abstract ([tiab]) terms to maximize retrieval of relevant literature. Searches covered January 1, 2015, to July 1, 2026, with emphasis on literature published from 2018 onward; earlier foundational sources were included selectively to provide context on pre-AI barriers to SUD screening and treatment. Full Boolean search strings appear in Supplementary File S1.

### 2.4 Eligibility criteria

Sources were included if they addressed development, evaluation, implementation, deployment, or acceptance of AI/ML tools relevant to alcohol, opioid, and cannabis use disorders screening or management; general AI-implementation-barriers literature; or pre-AI SUD screening and treatment barriers literature. Priority was given to empirical implementation or evaluation studies, algorithmic bias and fairness investigations, and implementation-science theoretical contributions. Purely technical ML performance studies were used selectively, primarily to characterize predictive-literature maturity by substance. Stimulant-use disorder was not included in the full synthesis because preliminary scoping searches identified only a small number of early-stage predictive-modeling studies and did not identify sufficient literature on implementation, deployment, or acceptance to support meaningful synthesis. This is noted as a scope limitation in Section 7.

### 2.5 Data synthesis

Evidence was synthesized iteratively against the four-domain hybrid framework, with themes cross-checked against both the general AI-implementation literature and the SUD-specific literature to identify convergence, SUD-specific amplification of general barriers, and barriers with no general-literature equivalent (Table 1).

**Table 1:** Summary of implementation domains, barriers, facilitators, and SUD-specific considerations.

Domain	Representative barriers	Representative facilitators	SUD-specific considerations	Illustrative evidence
Data and model	Limited external validation; underreported fairness auditing; incomplete EHR data continuity	Multimodal predeployment validation; bias mitigation algorithms; explainable outputs	Opioid literature most mature and most audited for bias; alcohol literature large but high risk of bias; cannabis literature early stage	(5, 19, 24–26)
Clinician and workflow	Added documentation burden; alert fatigue; liability concerns; low EHR-embedded automation	Preserved clinician oversight; clinician involvement in design	Chronic addiction-medicine workforce shortages amplify effort-expectancy barriers documented in SBIRT literature	(2–4, 16, 17)
Patient	Stigma-driven nondisclosure; unequal digital access; low trust in algorithmic decisions	Passive EHR-based screening reduces disclosure burden; personalized, trust-building message design	Trust burden is higher for longitudinal management tools than for one-time screening; equity risks compound with model- level bias	(9, 10, 22, 24)
System and regulatory	42 CFR Part 2 confidentiality constraints on data sharing; unclear reimbursement pathways; unsettled liability	Institutional leadership and infrastructure; clear governance for model monitoring and revalidation	SUD-specific confidentiality framework has no close parallel elsewhere in clinical AI; enforcement of 2024 final rule began only in 2026	(17, 18, 23)

EHR, electronic health record; SBIRT, Screening, Brief Intervention, and Referral to Treatment; SUD, substance use disorder.

## 3 What is distinct about alcohol, opioid, and cannabis use disorders as an AI implementation context

Four characteristics distinguish alcohol, opioid, and cannabis use disorders from most domains where AI implementation has been studied.

First, stigma shapes both disclosure and AI acceptance in ways general clinician-focused frameworks do not capture. Patients underreport substance use due to fear of judgment, legal consequence, job loss, or custody loss, and this extends to data-sharing willingness: a 2025 survey of 357 patients found stigma associated with reduced willingness to share SUD data outside one's primary treatment setting, while provider trust increased willingness (22). Beyond stigma, acceptance also depends on digital literacy, smartphone or internet access, comfort with algorithmic involvement in a personal domain of care, and trust in algorithmic decisions, factors that plausibly vary across the socioeconomic and racial/ethnic groups disproportionately affected by SUD and merit treatment as a distinct patient-level factor.

Second, alcohol, opioid, and cannabis use disorders care operates under 42 CFR Part 2, imposing confidentiality protections beyond HIPAA to reduce patients’ fear of legal, employment, or custodial consequences from seeking treatment (23). A 2024 final rule aligned Part 2 more closely with HIPAA, but HHS Office for Civil Rights enforcement began in 2026, an active regulatory transition with direct implications for pooling, sharing, and reusing SUD data for AI development and validation, with little parallel elsewhere in clinical AI.

Third, predictive evidence is uneven across substances in ways application-organized “AI in healthcare” reviews obscure. Alcohol use disorder has the largest predictive literature by volume, but this evidence base remains methodologically heterogeneous, with limited external validation and concerns about risk of bias (5). Despite limited real-world screening implementation, emerging management applications suggest progress toward clinical translation. Cannabis use disorder remains an earlier-stage evidence base. Opioid use disorder combines a mature predictive literature with the strongest real-world screening evidence (2, 19), but also the clearest documented algorithmic bias, including racial disparities in EHR-based opioid misuse prediction (24).

Fourth, management-focused AI carries different implementation demands than screening. Screening tools typically flag patients for clinician review; management applications may generate recovery-support content, deliver interventions, or inform medication-assisted treatment changes, interacting more directly with patients and demanding greater trust and perceived credibility. Emerging AI-generated recovery-support-messaging work reflects this recognition (10), a consideration not explicitly addressed in frameworks built around diagnostic and decision-support tools.

## 4 Barriers and facilitators across the four implementation domains

### 4.1 Data and model-level factors

The largest evidence body sits at the data and model level, revealing a tension between promising predictive performance and limited external validation, fairness assessment, and deployment readiness.

#### 4.1.1 Validation and generalizability

Few prediction models, across substances, have been externally validated on populations distinct from training cohorts, a persistent barrier throughout the broader AI-implementation literature (14, 16, 18). This matters more in SUD because treatment populations vary substantially across settings, regions, and payers.

#### 4.1.2 Bias and fairness: the most developed evidence in opioid use disorder

Although opioid use disorder has a smaller predictive literature by volume, its evidence base has progressed furthest across the methodological and translational dimensions assessed in this review, including external validation, deployment, and fairness evaluation. It was also the only substance for which we identified a deployed AI screening tool. An audit of an NLP opioid-misuse classifier found systematic racial-subgroup disadvantages and evaluated mitigation strategies (24). A 2024 fairness-aware framework reported meaningful reductions in racial disparities via an equalized-odds mitigation algorithm (25). These findings show algorithmic bias is measurable and modifiable rather than inevitable, but such evaluations remain uncommon: a 2025 Medicaid opioid acute-care model incorporated fairness analysis and reported improved equity but noted such assessment remains rare among newly published opioid models overall (26).

#### 4.1.3 Data completeness as an underlying mechanism

Foundational work identified missing, nonrandomly distributed EHR data as a general fairness threat (27); a manipulation study of an emergency department triage model found racial disparities in EHR missingness measurably degraded predictive performance for affected subgroups (28). This may be particularly relevant to SUD populations given fragmented care across systems, though it remains untested in addiction medicine specifically.

#### 4.1.4 Cannabis and alcohol: different maturity, different challenges

Cannabis prediction research remains earlier-stage and typically single-site, though a 2025 Bayesian model trained on a large national cohort achieved good discrimination and calibration for five-year cannabis use disorder risk across two external validation datasets (29). Cannabis models have not undergone opioid-level fairness scrutiny, reflecting limited deployment pressure rather than lower bias risk. Alcohol use disorder sits between the two: a 2022 systematic review of 70 studies found a methodologically diverse literature but most studies at moderate or high risk of bias due to limited external validation, without systematic fairness evaluation comparable to opioid research (5).

#### 4.1.5 Explainability as an emerging facilitator

Interpretability is increasingly treated as a precondition for clinical trust rather than a nice-to-have, echoing the “conclusiveness” theme in the broader clinician-AI-acceptance literature (16), though it remains more established as a design principle than a routine deployed feature.

### 4.2 Clinician and workflow-level factors

UTAUT's performance expectancy, effort expectancy, social influence, and facilitating conditions constructs organize this evidence (21), functioning ambivalently as barriers or facilitators depending on context (16). Of these four constructs, social influence, the effect of peer, institutional, and professional-society endorsement on clinician adoption, has not been directly measured in any SUD-specific AI implementation study identified in this review, a gap worth flagging given the potential role of institutional champions in the deployment successes described below.

#### 4.2.1 Performance expectancy and trust

Clinicians adopt AI tools more readily when they improve risk identification without displacing judgment. The opioid screening deployment preserved specialist review of every flagged case (2), consistent with clinician involvement in design and oversight as a recurring adoption facilitator across specialties (14, 17).

#### 4.2.2 Effort expectancy and workflow integration

Tools requiring substantial workflow change or added documentation burden face predictable resistance (14, 16). Pre-AI SBIRT research identified time pressure and competing priorities as primary screening barriers (3, 4). AI that automates detection from existing documentation, rather than requiring separate structured assessment, offers a meaningful facilitator, as in the opioid screening deployment (19).

#### 4.2.3 Facilitating conditions and organizational readiness

Deployment depends on EHR integration, technical support, and sustained leadership commitment (17); unclear responsibility for ongoing model monitoring is a recurring barrier (14, 18). SUD care, frequently delivered in resource-constrained community and safety-net settings, may be particularly vulnerable here relative to the academic medical centers where most deployments have occurred.

#### 4.2.4 Alert fatigue and clinician hesitancy

Alert fatigue was explicitly identified as an ongoing challenge in the opioid screening deployment even post-success (2). Legal and liability-related clinician hesitancy persists across specialties independent of demonstrated accuracy (16), potentially compounded in SUD by clinician awareness of bias risk for marginalized patients (24).

### 4.3 Patient-level factors

Patient-level factors operate differently for screening than for longitudinal management.

#### 4.3.1 Stigma and disclosure

Stigma-related legal, employment, and custodial concerns shape disclosure and data-sharing willingness, varying with perceived provider trustworthiness (22). Passive, EHR-based screening tools may reduce disclosure-related barriers relative to direct self-report, though this remains untested comparatively.

#### 4.3.2 Digital access and literacy

Management applications requiring sustained digital engagement over weeks or months (9, 10) introduce access barriers, smartphone ownership, reliable connectivity, digital comfort, absent from passive screening tools, unevenly distributed across groups disproportionately affected by SUD, risking widened rather than narrowed disparities if unaddressed.

#### 4.3.3 Trust in algorithmic decision making

Management applications’ repeated, direct patient interaction demands greater sustained trust. The AI-generated recovery-message protocol explicitly tests how message content affects engagement and perceived trustworthiness over 17 weeks (10).

#### 4.3.4 Equity considerations

Patient-level and data-level factors intersect: lower model accuracy for racial/ethnic minority patients (24) combined with lower baseline provider trust and data-sharing reluctance in the same populations (22) may compound rather than offset existing care disparities.

### 4.4 System and regulatory-level factors

#### 4.4.1 SUD-specific confidentiality regulation

42 CFR Part 2 imposes protections beyond HIPAA (23); the 2024 final rule aligned it more closely with HIPAA, but OCR enforcement only began in 2026, an active transition affecting which SUD data can be pooled for training and validation, with few parallels elsewhere in clinical AI.

#### 4.4.2 Reimbursement and sustainability

Sustained implementation requires clear reimbursement pathways and funded infrastructure. Both deployment cases operated within single, well-resourced institutional (University of Wisconsin Health) or commercial infrastructure, and neither addresses financial sustainability in less-resourced safety-net or community settings.

#### 4.4.3 Liability and governance

Clinician liability hesitancy (Section 4.2) is ultimately a governance question. Clear institutional protocols for AI-recommendation review and defined model-monitoring processes are consistent facilitators (17, 18); SUD-specific governance remains underdeveloped relative to fields such as radiology with more mature regulatory pathways.

## 5 Case studies of real-world deployment

### 5.1 Opioid use disorder screening

Among the deployment cases identified in this review, the clearest SUD screening success is a hospital-based deployment at University of Wisconsin Health. SMART-AI, a deep learning model identifying at-risk hospitalized patients from existing EHR data, underwent multimodal external validation including equitable-performance assessment across racial subgroups before deployment (19). In a prospective, pre–post quasi-experimental study spanning more than 51,000 hospitalized adults, the AI screener performed comparably to provider-only assessment in initiating specialist consultations. As a secondary outcome, AI screening was associated with 47 percent lower odds of 30-day readmission (odds ratio 0.53, 95% CI: 0.30–0.91, *P* = 0.02), at an incremental cost of approximately $6,801 per readmission avoided (2). Because this was a secondary outcome of a pre–post design in which secular trends were not fully controlled, it should be interpreted as an association rather than a demonstrated causal effect. Implementation preserved clinician oversight of every flagged case; authors identified alert fatigue and the need for broader cross-system validation as ongoing challenges. SMART-AI progressed from multimodal external validation (19) to prospective evaluation during live clinical deployment (2).

### 5.2 Alcohol use disorder relapse management

Previct Alcohol, a commercial eHealth platform combining breathalyzer monitoring with a machine learning relapse-prediction model, is, among the studies identified in this review, the strongest available example of sustained management deployment. The platform has operated for six years, though the AI relapse-prediction component was introduced later within that period. Analysis of observational real-world data spanning more than 500,000 patient-days, without randomization or a concurrent control arm, found AI-generated relapse predictions communicated to caregivers associated with a 9 percent reduction in short-duration relapse risk and an 18 percent reduction in longer-duration relapse risk relative to periods without caregiver notification (11). These figures derive from descriptive comparison of pre- and post-implementation periods; the primary source reports no confidence intervals or inferential statistics for these estimates, a limitation the authors themselves attribute to the real-world, nonrandomized character of the underlying data. Because the comparison is unadjusted and open to confounding by indication, and because relapse frequency varied across the observation period independently of the prediction tool, this evidence supports an association between the AI component and reduced relapse risk rather than a demonstrated causal benefit. Under this framework, Previct Alcohol is best classified as a live clinical deployment for which the cited evidence is a retrospective, observational analysis of real-world use (11) rather than a controlled or prospective validation study; it has not, to our knowledge, undergone external validation in the sense used elsewhere in this review.

### 5.3 A typology of management-focused applications

Relapse prediction, continuous monitoring, and AI-generated messaging are often grouped as “AI-supported management,” but differ substantially in directness and frequency of patient interaction. We propose a three-tier typology (Table 2).

**Table 2:** Typology of management-focused AI applications in SUD care, by tier of patient interaction.

Tier	Definition	Patient interaction	Trust burden	Example
Tier 1: Passive risk stratification	Generates a retrospective or point-in-time risk score from data already produced by the patient	None; output directed to the clinical team, not the patient	Low	(8)
Tier 2: Active monitoring and alerting	Collects data continuously or repeatedly and generates ongoing risk signals during treatment	Ongoing but largely passive (continuous monitoring, repeated self-report)	Moderate	(9, 11)
Tier 3: AI-driven therapeutic interaction	Generates content delivered directly to the patient to influence behavior or engagement	Direct and repeated; patient reads and is expected to act on AI-generated content	High	(10)

Trust burden refers to the degree of sustained patient trust and engagement required for the tool to function as intended, not to the tool's predictive accuracy.

Tier 1: Passive Risk Stratification generates a retrospective or point-in-time risk estimate without ongoing patient-facing interaction; the social-media-language digital phenotyping model exemplifies this tier (8).

Tier 2: Active Monitoring and Alerting collects data continuously or repeatedly, generating ongoing risk signals. Previct Alcohol (11) and the smartphone EMA opioid-relapse model (9) operate here.

Tier 3: AI-Driven Therapeutic Interaction delivers content directly to patients to influence behavior; AI-generated recovery-support messaging occupies this tier (10).

Trust burden escalates from Tier 1 (low; data already collected for other purposes) through Tier 2 (moderate; sustained tolerance of passive monitoring) to Tier 3 (high; patients must trust and act on AI-generated content directly), the tier current research is only beginning to evaluate empirically.

### 5.4 Cannabis use disorder and the limits of current evidence

Cannabis use disorder illustrates the opposite translational extreme. Predictive research remains comparatively early in volume, though not uniformly limited in rigor; a 2025 Bayesian model trained on a large national cohort and externally validated on two independent datasets achieved good discrimination and calibration for five-year risk (29). No cannabis-specific tool identified here has undergone deployment comparable to the opioid or alcohol examples, likely reflecting a smaller, more recent literature, less regulatory urgency than the opioid crisis, and the absence of a champion institution comparable to SMART-AI's or Previct Alcohol's developers, a translational-pipeline gap rather than evidence AI is inherently less applicable to cannabis.

## 6 Discussion

### 6.1 Synthesis across domains

Both case studies showed favorable conditions across multiple implementation domains, although the nature and strength of the evidence differed. SMART-AI has documented implementation-outcome evidence, preserved clinician oversight, prospective evaluation within live clinical workflows, and alert fatigue as an ongoing implementation barrier (2, 19), alongside evidence of clinical effectiveness through reduced 30-day readmission odds and reported assessment of algorithmic fairness and bias (24). Previct Alcohol has evidence of clinical effectiveness and sustained real-world use over multiple years, but comparable implementation-outcome and fairness assessments were not identified in the cited source (11). We therefore distinguish clinical effectiveness from implementation success throughout this review.

This interpretation is consistent with the broader literature emphasizing multilevel determinants of AI adoption (14, 18) and suggests that implementation in SUD care may require attention to multiple interacting factors, including clinical workflow, clinician engagement, stigma, and regulatory considerations.

### 6.2 SUD-specific considerations revisited

Three features recur throughout: stigma operates as both a patient-level disclosure barrier (22) and an amplifier of algorithmic bias consequences, since inequitable performance for marginalized groups (24) compounds pre-existing distrust; the evolving 42 CFR Part 2 environment constrains the cross-system data sharing needed for external validation; and management applications face a trust burden that, per the Section 5.3 typology, escalates with directness and frequency of patient interaction rather than with predictive accuracy alone.

### 6.3 Research and translation agenda

Four measurable priorities emerge. First, prioritize external validation in health systems with high Medicaid-insured or uninsured populations: a specific target is validating an existing SUD model in at least two health systems where Medicaid enrollees constitute 30 percent or more of the study population, reporting calibration slope and intercept by site alongside discrimination metrics. Second, make fairness auditing standard rather than exceptional, extending opioid-research practices (24–26) to alcohol and cannabis: a specific target is a minimum standard of at least three complementary fairness metrics prior to deployment. Third, extend implementation research beyond academic medical centers into resource-constrained community and safety-net settings: a specific target is prospective evaluation of an existing validated tool in at least two Federally Qualified Health Centers or opioid treatment programs within three to five years, with facilitating conditions measured explicitly. Fourth, as 42 CFR Part 2 enforcement proceeds, evaluate its effect on cross-system data sharing: a specific target is comparing the number and scale of multi-site SUD AI validation studies initiated in the three years before versus after the 2026 enforcement date.

## 7 Limitations

This narrative review does not include a PRISMA flow diagram, formal risk-of-bias assessment, or protocol registration. Although study identification was guided by a structured search strategy, study selection and synthesis involved author judgment rather than a fully reproducible systematic screening process. Accordingly, comparative and superlative statements throughout the review (e.g., regarding which substance has the largest or most mature literature, or which deployment represents the strongest example) should be interpreted as describing the literature identified through our search strategy rather than the entire evidence base.

The review is scoped to alcohol, opioid, and cannabis use disorders. Stimulant-use disorders were not included in the full synthesis because preliminary scoping searches did not identify sufficient implementation-relevant literature to support meaningful comparison with the three substances reviewed. Conclusions should therefore not be extended to stimulant-use disorders.

Both deployment case studies originate from well-resourced U.S. institutional or commercial settings, which may limit generalizability to other health systems, payer environments, and regulatory contexts. SUD-specific AI implementation literature also remains limited relative to the broader implementation literature; consequently, some domain-level conclusions, particularly at the patient and system levels, draw partly on evidence from adjacent fields. Finally, because this field is evolving rapidly, including ongoing protocols and preprints without reported outcomes (10), some evidence characterized as early-stage may have progressed by the time of publication.

## 8 Conclusion

AI-based tools for SUD screening and management show strong predictive performance across alcohol, opioid, and cannabis use disorders, but real-world implementation remains confined to a narrow set of applications. Among the studies identified in this review, opioid use disorder provides the strongest AI-assisted screening evidence, alcohol use disorder provides the strongest AI-supported management evidence, and no substance has yet demonstrated success across both. Successful implementation appears to require alignment across data and model, clinician and workflow, patient, and system and regulatory domains simultaneously, a bar met so far only within well-resourced institutional settings. Closing the gap between AI's demonstrated predictive capability and its limited adoption in routine SUD care will require external validation across diverse settings, routine fairness auditing, implementation research extended into community and safety-net treatment settings, and continued attention to the evolving regulatory environment governing SUD data.
