# Supplementary material for: Artificial intelligence for alcohol, opioid, and cannabis use disorders screening and management: a narrative review of barriers and facilitators to clinical implementation
Source: Front Digit Health. 2026 Sep 21;8:1958597. doi: 10.3389/fdgth.2026.1958597 (PMC13635661; doi:10.3389/fdgth.2026.1958597)
Supplement: Supplementary file 1 [file Supplementaryfile1.docx]

# **Supplementary File S1. Database Search Strings**

**PubMed:** ("Artificial Intelligence"[Mesh] OR "Machine Learning"[Mesh] OR "Deep Learning"[Mesh] OR "artificial intelligence"[tiab] OR "machine learning"[tiab] OR "deep learning"[tiab] OR "natural language processing"[tiab]) AND ("Substance-Related Disorders"[Mesh] OR "Opioid-Related Disorders"[Mesh] OR "Alcoholism"[Mesh] OR "substance use disorder"[tiab] OR "substance use disorders"[tiab] OR opioid[tiab] OR "alcohol use disorder"[tiab] OR "cannabis use disorder"[tiab] OR addiction[tiab]) AND (implementation[tiab] OR barrier[tiab] OR facilitator[tiab] OR adoption[tiab] OR acceptance[tiab] OR deployment[tiab] OR "clinical decision support"[tiab])

**Embase:** ('artificial intelligence':ti,ab OR 'machine learning':ti,ab OR 'deep learning':ti,ab OR 'natural language processing':ti,ab) AND ('drug dependence'/exp OR 'alcoholism'/exp OR 'substance use disorder':ti,ab OR 'substance use disorders':ti,ab OR opioid:ti,ab OR 'alcohol use disorder':ti,ab OR 'cannabis use disorder':ti,ab OR addiction:ti,ab) AND (implementation:ti,ab OR barrier:ti,ab OR facilitator:ti,ab OR adoption:ti,ab OR acceptance:ti,ab OR deployment:ti,ab OR 'clinical decision support':ti,ab)

**Web of Science:** TS=(("Artificial Intelligence" OR "Machine Learning" OR "Deep Learning" OR "natural language processing") AND ("substance use disorder" OR "substance use disorders" OR opioid OR "alcohol use disorder" OR "cannabis use disorder" OR addiction) AND (implementation OR barrier OR facilitator OR adoption OR acceptance OR deployment OR "clinical decision support"))

**PsycINFO:** (exp Artificial Intelligence/ OR exp Machine Learning/ OR "artificial intelligence".ti,ab. OR "machine learning".ti,ab. OR "deep learning".ti,ab. OR "natural language processing".ti,ab.) AND (exp Substance Use Disorders/ OR exp Drug Abuse/ OR exp Alcoholism/ OR "substance use disorder".ti,ab. OR "substance use disorders".ti,ab. OR opioid.ti,ab. OR "alcohol use disorder".ti,ab. OR "cannabis use disorder".ti,ab. OR addiction.ti,ab.) AND (implementation.ti,ab. OR barrier.ti,ab. OR facilitator.ti,ab. OR adoption.ti,ab. OR acceptance.ti,ab. OR deployment.ti,ab. OR "clinical decision support".ti,ab.)

**medRxiv:** ("artificial intelligence" OR "machine learning" OR "deep learning") AND ("substance use disorder" OR opioid OR "alcohol use disorder" OR "cannabis use disorder" OR addiction) AND (implementation OR barrier OR facilitator OR adoption OR acceptance OR deployment)

**arXiv:** abs:("artificial intelligence" OR "machine learning" OR "deep learning") AND abs:("substance use disorder" OR opioid OR alcohol OR cannabis OR addiction) AND abs:(implementation OR barrier OR facilitator OR adoption OR acceptance OR deployment)

Searches were limited to English-language records, covering January 1, 2015 to July 1, 2026. No study-design filter was applied at the search stage; design-based prioritization occurred during eligibility screening (Section 2.4). Citation tracking and targeted searches were also used to identify relevant literature not captured by the database searches, including pre-AI literature on SUD screening barriers.
